# Patterns of Alcohol Use in Hispanic Individuals with TBI over the Ten Years Post-Injury: A Model Systems Study

**DOI:** 10.3390/jpm14010105

**Published:** 2024-01-18

**Authors:** Juan Carlos Arango-Lasprilla, Jack D. Watson, Miriam J. Rodríguez, Daniela Ramos-Usuga, Paul B. Perrin

**Affiliations:** 1 Department of Psychology, Virginia Commonwealth University, Richmond, VA 23284, USA; watsonjd4@vcu.edu; 2 School of Public Health, Department of Health and Wellness Design, Indiana University, Bloomington, IN 47408, USA; mijrodri@iu.edu; 3 Biomedical Research Doctorate Program, Department of Cell Biology and Histology, University of the Basque Country (UPV/EHU), 48940 Leioa, Spain; daniela.ramos.usuga88@gmail.com; 4 School of Data Science, Department of Psychology, University of Virginia, Charlottesville, VA 22904, USA; 5 Central Virginia Veterans Affairs Health Care System, Richmond, VA 23249, USA

**Keywords:** alcohol, substance use, traumatic brain injury, hispanics

## Abstract

The study’s aim was to examine alcohol consumption patterns and predictors of consumption across time among Hispanics with traumatic brain injury (TBI) in the U.S. within ten years post-injury. This longitudinal cohort study included 1342 Hispanic individuals (77.6% males) from the multi-site, longitudinal TBI Model Systems (TBIMS) database. The main outcome measures were consumption information, demographic, and injury characteristics. Across the full sample, alcohol consumption variables generally demonstrated quadratic movement characterized by an initial increase followed by a plateau or slight decrease over the ten years post-injury. The predictors of higher consumption were being men, single, with a history of excessive alcohol use, with a nonviolent mechanism of injury, shorter duration of PTA, and higher levels of education. Participants had a greater number of 5+ drinks/episode occurrences in the past month if they were men and had had a greater number of 5+ drinks/episode occurrences in the month before injury. There was no differential change in alcohol consumption over time as a function of these predictors. This study identified a profile of at-risk Hispanics with TBI for increased alcohol consumption. These individuals should be identified and targeted for early evidence-based alcohol intervention after TBI when results might be most favorable.

## 1. Introduction

About 5.3 million Americans live with some form of disability due to traumatic brain injury (TBI; [1]). Many of these individuals are left with physical, emotional, and cognitive problems. In the cognitive domain, a lack of self-regulation of behavior is one of the most frequent problems observed, and this has an impact on personal, social, work, and family life [2]. Due to these sequelae, people with TBI are more likely than the general population to present socially disinhibited behaviors [3] and risky behaviors such as inappropriate and/or risky sexual practices [3,4], violent behavior, criminal acts [2,3], and the use of alcohol and illicit drugs [5]. 

In relation to alcohol consumption in people with TBI, some studies have examined alcohol consumption as a risk factor for sustaining a TBI, while others have focused on determining to what extent the history of TBI is a risk factor for alcohol problems or examining the pattern of consumption after the injury. In reference to the first line of research, alcohol intoxication is an important risk factor for TBI [6], with elevated alcohol levels in 30–40% of cases [7,8,9,10,11]. In fact, more than half of individuals with TBI admitted for rehabilitation meet the criteria for Alcohol Use Disorder (AUD) [12]. In addition, alcohol misuse and/or AUD are associated with higher mortality, and, in survivors, with a higher probability of a second TBI, longer and more complicated hospital stays, and worse cognitive functioning [13,14]. The latter is explained, in part, by the fact that prolonged alcohol consumption causes structural and functional damage to the brain, known as alcohol-related brain damage (ARBD), and those with a history of alcohol misuse usually have a worse prognosis after injury [15,16]. Regarding the second line of research, findings are inconsistent. Many of the studies agree that the pattern of consumption after injury in individuals with previous alcohol problems is characterized by a decrease in consumption in the first months after TBI and a progressive increase to pre-injury levels over the following years [7,12,17,18,19,20]. For example, in a sample of active duty military and veterans with TBI, Steffen-Allen and colleagues [21] found this same pattern of use in moderate and severe TBI cases. However, participants with mild TBI showed an opposite pattern, with an increase in consumption in the initial stages followed by a decrease. Other authors have found that alcohol misuse is more prevalent among individuals with TBI compared to healthy individuals [8,22], or that even some abstinent individuals develop problems with alcohol consumption after injury [13]. Furthermore, studies conducted with children and adolescents with TBI have found that an early brain injury impairs neurodevelopment and may be an independent risk factor for the development of alcohol problems in adulthood [23,24,25,26]. However, in a recent systematic review, Olsen and Corriga [27] conclude that although there is a high incidence of alcohol consumption in individuals with TBI, there is still insufficient knowledge to establish causality. Despite these discrepancies in the literature, some risk factors have been identified that seem to facilitate alcohol consumption or misuse in people with TBI, such as being male [18,20,28,29], of a younger age [17,20,28], engaging in other substance use before injury [12,18,20,28], intoxication at the time of injury [17], symptoms of depression after injury [28], better physical functioning [28], and not being in a romantic relationship [17]. 

With regard to race/ethnicity, it has been widely shown that minorities, particularly Hispanics, tend to have worse functional, cognitive, and emotional outcomes [30,31,32,33], as well as worse social reintegration [32,34], work outcomes [32,34,35], and marital outcomes [32] after TBI. Presumably, therefore, a worse prognosis may be associated with a higher chance of developing alcohol problems in this population. However, to date, the relationship between race/ethnicity and alcohol consumption in the long term after TBI has not been investigated. 

Some possible explanations for this may be related to the following facts: (a) many of the studies on alcohol and TBI do not include the race/ethnicity variable in the analyses [7,12,17,21]; (b) those that include it only report it in the descriptive results [8,18,19,20,22]; and finally (c) some investigations have been cross-sectional and carried out only during the first years after TBI [8,22]. For all of these reasons, it is important to study alcohol consumption in minority groups in the United States, especially in Hispanics, since they represent 18.7% of the total population [36]. Therefore, the objective of the present study is to explore the patterns of alcohol consumption of the Hispanic population with TBI in the United States during the first 10 years after the injury and to determine the predictive variables associated with this consumption.

## 2. Materials and Methods

### 2.1. Participants

This longitudinal study included 1342 Hispanic individuals from the U.S. Traumatic Brain Injury Model Systems (TBIMS) database. Each collaborating center’s Institutional Review Board provided human subjects’ approval. Participants provided written informed consent or their proxy/legal guardian if applicable. The study was conducted in accordance with the Declaration of Helsinki.

Participants enrolled after TBI and during inpatient rehabilitation at a TBIMS hospital. The participants in the current study sustained their TBI between 1991 and 2016. The eligibility criteria were the following: 16 years of age or older at injury, received medical care within 72 h after injury and inpatient rehabilitation at a TBIMS hospital, and have sustained a TBI via external mechanical force resulting (a) loss of consciousness greater than 30 min, (b) Glasgow Coma Scale (GCS) score at emergency department admission less than 13, (c) intracranial abnormality on neuroimaging due to trauma, or (d) posttraumatic amnesia (PTA) greater than 24 h. 

A total of 1792 Hispanic individuals were in the TBIMS dataset; 1342 were included in this study if they had at least one data point at a follow-up data collection (years 1, 2, 5, or 10) regarding the average number of drinks consumed per episode of drinking in the previous month (henceforth referred to as “average number of drinks per episode”) and one complete data point regarding the number of times when five or more drinks were consumed in a single episode in the previous month (further referred to as “five or more drinks in the previous month”). In the retained sample, 1151 people had complete data for the average number of drinks per episode at year 1; 967 at year 2; 560 at year 5; and 267 at year 10. For five or more drinks in the previous month, 1153 people had complete data at year 1; 967 at year 2; 558 at year 5; and 267 at year 10. Demographic data appear in Table 1. The full information maximum likelihood (FIML) estimation was used to include participants with missing data (as long as they had at least one outcome variable for a particular analysis); therefore, differential attrition, if present, would not bear on the conclusions drawn.

### 2.2. Variable Recoding for Analyses

Both the baseline variable for the average number of drinks per episode and the baseline variable for five or more drinks in the previous month were given a reference point of 0. Romantic relationship status was coded with “married” as “1” and all other options like “single”, “divorced”, etc., were coded as “0”. Employment at injury was coded with “competitively employed” as “1” and all other categories including “full-time student”, “homemaker”, “retired”, etc., as “0”. Insurance type was dichotomized with “private insurance”, “worker’s compensation”, “private insurance: other”, “HMO”, “auto insurance”, “PPO”, and “TRICARE/TRIWEST” coded as “1”, while other options including “Medicare”, “Medicaid”, “self-pay”, etc., were coded as “0”. The language spoken at home was dichotomized such that “English” was “1” and “Spanish” and “Other Language” were “0”. The cause of injury was recoded to reflect violent vs. non-violent mechanisms of injury. The mechanisms of “gunshot wound”, “assaults with blunt instrument”, and “other violence” were “1”, while other mechanisms like “water sports”, “motor vehicle”, etc., were coded as “0”. Country of origin and sex were already dichotomized, and education was retained as continuous.

### 2.3. Data Analysis

Hierarchical linear modeling (HLM) examined the baseline predictors of the average number of drinks per episode and five or more drinks in the previous month across 1, 2, 5, and 10 years post-injury. The initial models included time, time*time, and time*time*time as predictors to determine whether the average number of drinks per episode and five or more drinks in the previous month scores had linear, quadratic, or cubic curvature. A decrease in -2 log likelihood [-2LL] between models of 3.84 reflects a significant improvement from the previous model. In the main effects HLMs, predictors were entered as fixed effects after centering or recoding with a reference point of 0, as well as time (and a higher-order time, depending on the curvature). The first main effects HLMs examined whether the trajectories of the average number of drinks per episode and five or more drinks in the previous month over years 1, 2, 5, and 10 could be predicted by the following baseline injury characteristics and demographics: time (0 = one year, 1 = 2 years, 4 = 5 years, and 9 = 10 years), age, sex, marital status, length of PTA, education level, employment at injury, annual earnings, insurance type, country of origin, language spoken at home, and violence as a cause of injury. Additionally, for the HLM examining the average number of drinks per episode, the baseline variable for the average number of drinks per episode in the month before injury was included. Similarly, for five or more drinks, the respective pre-injury baseline variable was also included. To test for differences in the slope as a function of the predictor, a final set of HLMs was run with each of the significant predictors from the main effects models, time (and higher-order time if applicable), and the interactions between time (or the higher-order terms) and the significant predictor.

## 3. Results

### 3.1. Curvature Models for Average Number of Drinks per Episode

The -2LL of the model with linear time was 12,177.99. The -2LL of the model with quadratic time was 12,172.05, a drop of greater than 3.84 χ^2^ points. The cubic time was 12,169.34 and less than 3.84 χ^2^ points. Therefore, the quadratic trajectories of the average number of drinks per episode was a better fit to the data than either linear or cubic time.

### 3.2. Full Model for Average Number of Drinks per Episode

**Figure 1:**
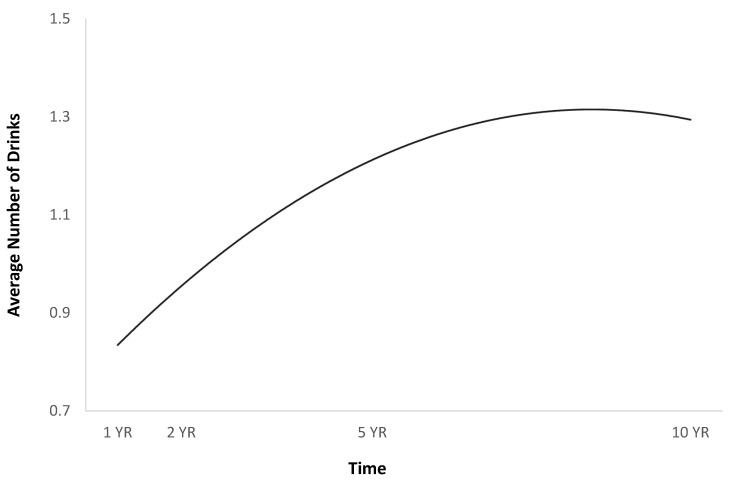
Average number of drinks per episode over time.

**Figure 2:**
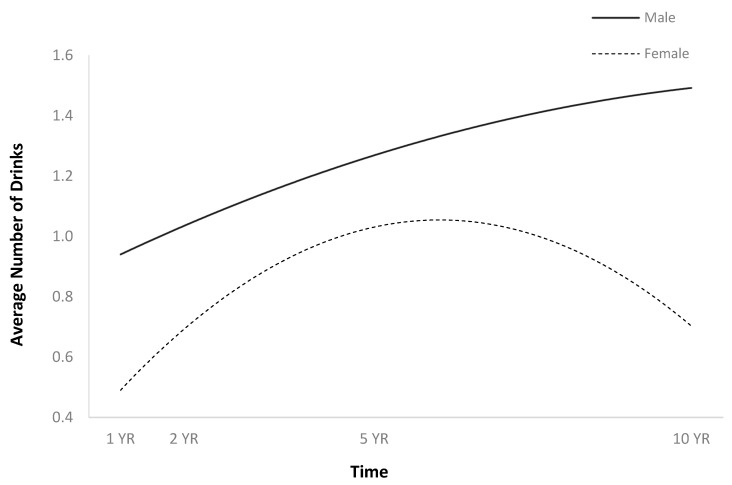
Main effect of sex on average number of drinks per episode.

**Figure 3:**
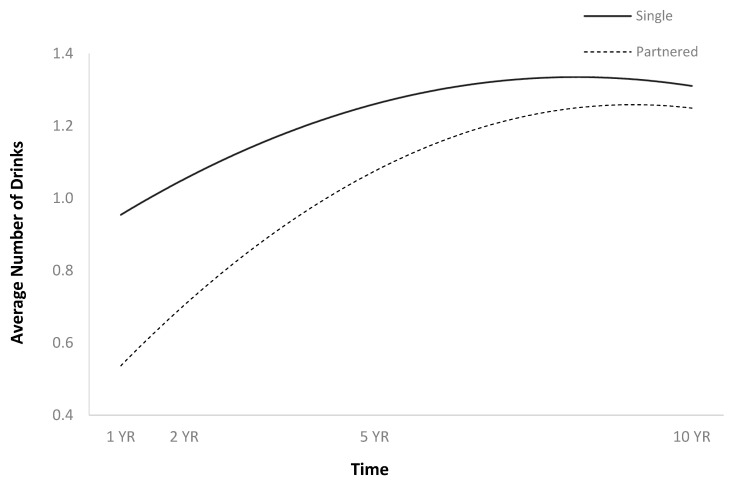
Main effect of romantic relationship status on average number of drinks per episode.

**Figure 4:**
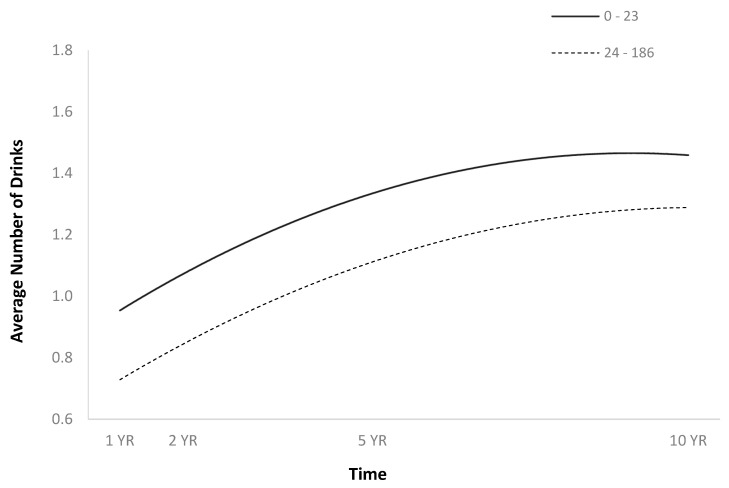
Main effect of PTA on average number of drinks per episode. For graphing purposes, time in PTA was median split.

**Figure 5:**
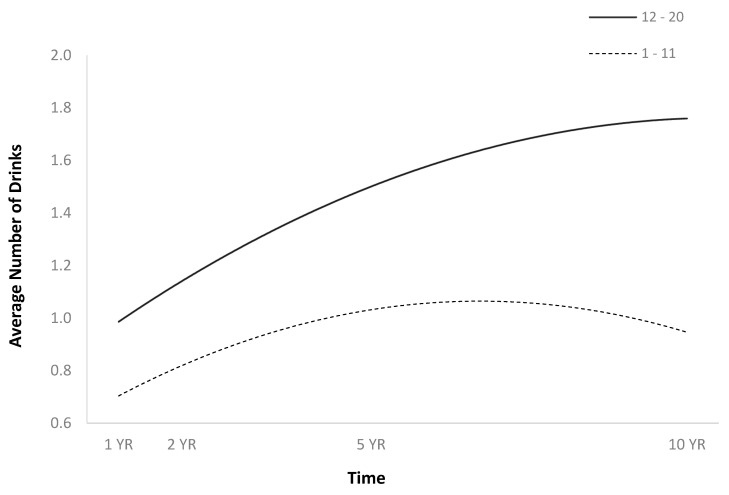
Main effect of education on average number of drinks per episode. For graphing purposes, years of education was median split.

**Figure 6:**
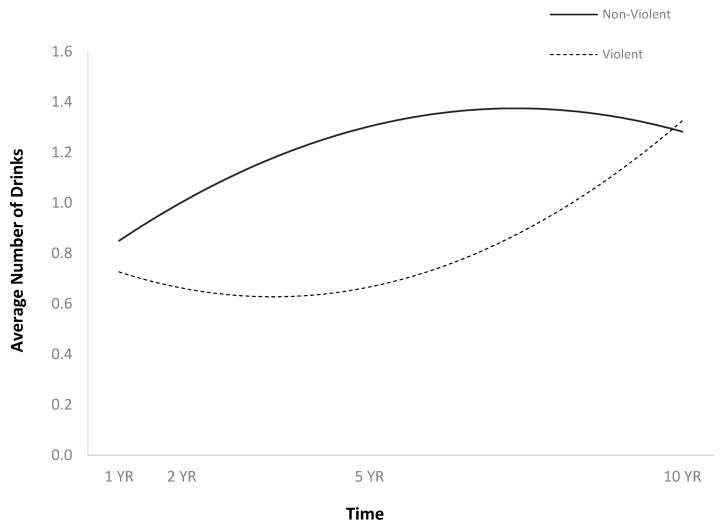
Main effect of violent cause of injury on average number of drinks per episode.

**Figure 7:**
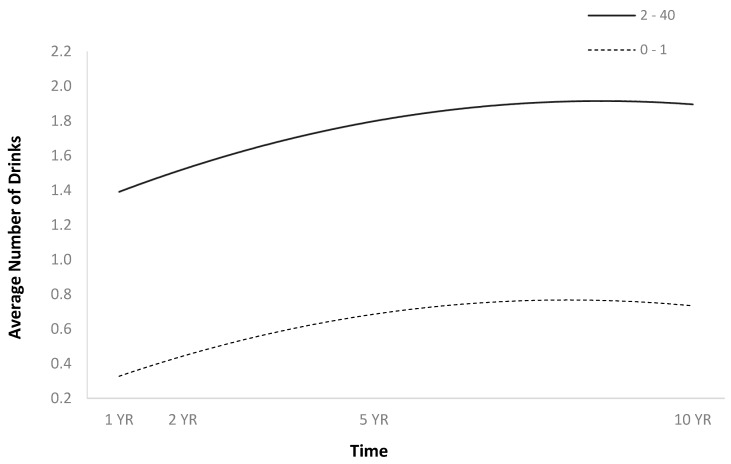
Main effect of average number of drinks per episode before injury on average number of drinks per episode over time. Note: For graphing purposes, the average number of drinks per episode before injury was median split.

The full HLM examined whether the quadratic trajectories over time of the average number of drinks per episode could be predicted by baseline injury characteristics and demographics (Table 2). The quadratic trend over time of the average number of drinks per episode demonstrated an initial increase followed by a plateau or slight decrease (Figure 1). Participants were more likely to consume a greater number of drinks per episode over time if they were men (Figure 2), single (Figure 3), had spent less time in PTA (Figure 4), had higher levels of education (Figure 5), had experienced a nonviolent mechanism of injury (Figure 6), and had consumed a greater number of drinks per episode before injury (Figure 7).

### 3.3. Models with Quadratic Time Interactions for Average Number of Drinks

Six HLMs examined whether the quadratic trajectories of the average number of drinks per episode could be predicted by the prior significant predictors and their interactions with time (Table 3). There was no significant interaction term for any of the previously significant predictors, suggesting no differential movement over time as a function of these variables.

### 3.4. Curvature Models for Five or More Drinks Consumed per Episode in the Previous Month

The -2LL of the model with linear time was 13,319.03. The -2LL of the model with quadratic time was 13,315.19, a drop of greater than 3.84 χ^2^ points. The cubic time was 13,315.18 and less than 3.84 χ^2^ points. Therefore, the quadratic movement of trajectories of five or more drinks per episode in the previous month was a better fit to the data than the linear or cubic time.

### 3.5. Full Model for Five or More Drinks per Episode in the Previous Month

**Figure 8:**
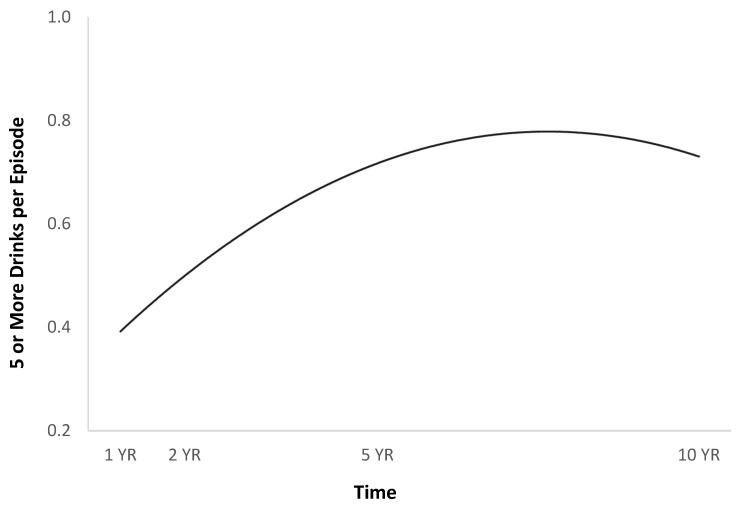
Times consumed five or more drinks per episode in the previous month over time.

**Figure 9:**
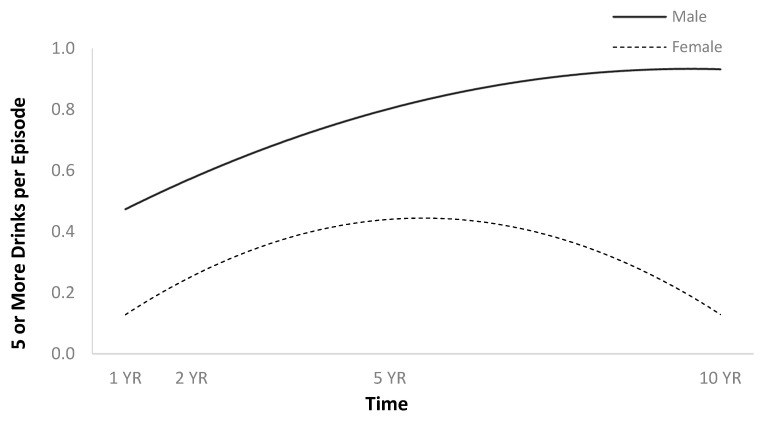
Main effect of sex on five or more drinks per episode in the previous month.

**Figure 10:**
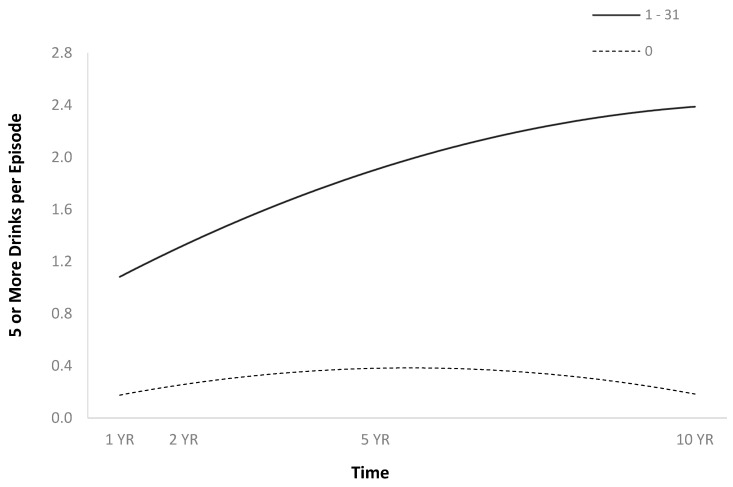
Main effect of number of times consumed five or more drinks per episode in the month before injury on five or more drinks per episode in the previous month. Note: For graphing purposes, the number of times consumed five or more drinks per episode in the month before injury was median split.

The full HLM examined whether the quadratic trajectories over time of five or more drinks per episode in the previous month could be predicted by baseline injury characteristics and demographics (Table 2). The quadratic trend for five or more drinks per episode in the previous month showed an initial increase, then a plateau or slight decrease (Figure 8). Participants had more episodes in the past month when they had consumed five or more drinks if they were men (Figure 9), and had had more episodes in the month before injury when they had consumed five or more drinks (Figure 10).

### 3.6. Models with Quadratic Time Interactions for Five or More Drinks per Episode in the Previous Month

Two HLMs examined whether the quadratic trajectories of five or more drinks per episode in the previous month could be predicted by the prior significant predictors and their interactions with time (Table 3). There was no significant interaction term for any of the prior significant predictors, suggesting no differential movement over time as a function of these variables.

## 4. Discussion

The aim of the current study was to examine alcohol consumption patterns among Hispanic individuals with TBI within ten years post-injury. The results indicated that Hispanics with TBI initially increased their alcohol intake after injury, and then plateaued or slightly decreased over the trajectory of ten years. Furthermore, the results of HLMs examining the predictive relationships of demographic variables indicated that single males with a shorter duration of PTA, higher levels of education, nonviolent mechanisms of injury, and a history of consumption of greater amounts of alcohol before injury were more likely to consume more alcohol per episode post-TBI. The findings contribute to a very limited descriptive literature on alcohol consumption patterns for Hispanic TBI populations.

Previous research on alcohol use post-TBI has historically either included predominately White samples, or ethnicity was not addressed or considered in their methodology. A significant decrease in alcohol use a few months post-TBI has been observed in these studies with an increase in alcohol intake by the end of the first year and individuals with mild TBI being at higher risk. These studies further indicate that pre-injury alcohol use is predictive of post-injury use [7,9,10,12,17,18,19,37]. The current findings indicated that alcohol use patterns post-TBI among Hispanics are similar to these previous findings in that being male and having a history of high alcohol consumption prior to the injury are predictive of future alcohol use post-TBI. The findings showed that shorter duration of PTA and non-violent mechanisms of injury, which is often indicative of milder TBI, are predictive of increased alcohol use among Hispanics.

The findings Identify additional factors that contribute to increased alcohol intake among Hispanics with TBI, such as higher levels of education and single relationship status, and further describe alcohol use patterns longitudinally over 10 years. These findings identified that among Hispanic individuals, post-TBI binge drinking initially increases and then stabilizes over time (Figure 1). Previous studies examining longitudinal alcohol use post-TBI among other samples have identified similar patterns and have noted an initial decrease in use followed by an increase of up to five years post-TBI [7,12,17,18,19,20,37]. Our findings indicate that after five years post-TBI, alcohol use slightly decreased among this Hispanic sample.

The current findings are consistent with the previous literature in identifying sex differences in substance abuse disorders among predominately White TBI samples. Males have been identified as being more likely to binge drink and more likely to return to high levels of drinking after TBI [8,29]. The current findings indicate that this pattern is consistent among Hispanic individuals, with single males being more likely than females to consume more alcohol per episode post-TBI. Previous studies among predominately White samples have identified age as a significant predictor of alcohol use post-TBI, with younger males being at greater risk [8,17,28]. The current findings indicate that among Hispanic TBI individuals, age was not a significant predictor. 

Similar to this study’s methodology, the previous literature has examined associations between binge drinking as defined by five or more drinks per episode on the same occasion during the previous month among other populations [22,28]. Adams and colleagues [22] identified that among active duty military personnel, past month frequent binge drinking was associated with obtaining a TBI, indicating that it was a significant risk factor. The current results indicate that in addition to drinking behavior prior to injury being a known risk factor for obtaining a TBI, among Hispanic individuals, it is additionally associated with further alcohol consumption in the years following TBI. 

There are important implications to studying alcohol use patterns among Hispanics with TBI. Excessive alcohol use is a public health concern in that it affects health and social functioning and is itself associated with structural and functional abnormalities in the brain and poorer recovery post-TBI [14,15,16,23]. More severe consequences can lead to other neurological disorders such as Wernicke-Korsakoff syndrome. Excessive alcohol use after TBI exacerbates the risk of further damage to the brain and future neurological problems and disorders. Therefore, it is important to identify individuals who are at greater risk for alcohol use following TBI and target these individuals with evidence-based alcohol reduction interventions. The current study identified a profile of at-risk Hispanic individuals with TBI to include males with high school education and above and single marital status who have a history of alcohol use and mild TBI. These individuals should be the focus of interventions to prevent future alcohol use. These interventions may include educational programs, counseling services, or support groups. There is evidence to support that alcohol interventions are most effective among Latino males when they are culturally tailored [38], and the use of motivational interviewing techniques are especially effective for treating heavy drinking among Hispanics [39].

Our findings indicate that age is not a risk factor among this Hispanic sample. This finding is particularly significant as alcohol dependence among older individuals is often unrecognized and undertreated. The implications of our results show that among Hispanics, alcohol reduction interventions can be beneficial at all ages. Future research should examine other barriers to seeking and receiving alcohol treatment across the lifespan and interventions should be tailored to address these barriers. For example, interventions should be tailored to address age-specific challenges that are experienced at various life stages, such as mobility limitations among older adults, and peer pressure among younger adults. Other barriers that should be taken into consideration include cultural and socio-economic factors that might limit accessibility to alcohol interventions. Interventions should be scalable, accessible to individuals from low socio-economic backgrounds (i.e., local and affordable), and available in Spanish. It is additionally important for health care professionals and staff to be of similar ethnicity to optimize an effective response to treatment among Hispanics [40]. 

The results of this study should be integrated into clinical practice by incorporating valid assessment tools to identify alcohol consumption post-TBI among Hispanics. Some assessment tools that have been validated among Hispanics in the U.S. include the CAGE (4M, [41]) and the Alcohol Use Disorders Identification Test-Consumption (AUDIT-C, [42]). Additionally, these results may inform policy makers to consider, when developing culturally competent services and policy measures, how to best reduce alcohol consumption and harm among Hispanic individuals post-TBI. 

This study identified a plateau or slight decrease in alcohol use after five years. Future studies should examine which factors contribute towards this plateau or decrease and how these factors can be incorporated into intervention programs to promote decreases in alcohol use earlier. Factors like age, employment, marital status, education, and other socioeconomic factors should be examined as possible predictors of decreases in alcohol use over time. There has been an interest among researchers to examine TBI as an independent risk factor for future alcohol use disorders [11,24,25]. The current study did not examine this association, but this is a potential future direction to examine among Hispanic individuals.

A strength of this study was the use of a large dataset and a large sample of Hispanics with TBI. To our knowledge, this is the first study that has examined alcohol use patterns post-TBI in a large sample of Hispanic participants. 

### Limitations

Some limitations include the lack of inclusion of other variables, which could be associated with increased alcohol use post-TBI, such as a history of previous TBIs or immigration status. Future studies should examine these factors as well as compare alcohol use patterns among Hispanics with TBI, White samples, and other racial/ethnic groups. Additionally, in the current study, the language spoken at home was dichotomized as a predictor into English vs. Spanish/Other Language. There are many communities in Latin America that speak indigenous languages such as Quechua, Miskito, etc., and Hispanic-identifying individuals in the US could easily speak these languages at home. Unfortunately, the TBIMS study does collect data on other languages besides English, Spanish, and “Other Language”. While the dichotomy in the current study was performed based on a theory of acculturation—that is, those who speak English at home (as opposed to Spanish or an Other Language) likely have a greater degree of acculturation—future research would benefit from collecting data on specific language(s) spoken and other indices of acculturation. In addition to language differences, the Hispanic population is culturally, socially, and economically diverse, which can influence alcohol consumption patterns. In the present study, the differences between subgroups of Hispanics were not studied, so there could be differences that were not evidenced in this research. Future studies should study the patterns of alcohol consumption taking into account the diversity of this population, such as the country of origin.

## 5. Conclusions

In conclusion, this study was one of the first to examine alcohol use patterns among a large Hispanic sample post-TBI, longitudinally over 10 years. The findings identified a profile of at-risk individuals to include single Hispanic male patients with a history of excessive alcohol use, with a nonviolent mechanism of injury and shorter duration of PTA (indicative of milder TBI), and higher levels of education. These individuals may be at risk of increased alcohol use up to ten years post-TBI. These individuals should be identified and targeted for early alcohol intervention after TBI when the results might be most favorable. Furthermore, the results indicate that these interventions might apply equally well at all ages among this particular population.

## Figures and Tables

**Table 1:** Sample baseline characteristics.

Characteristics	(*N* = 1342)
Age, *M* (*SD*)	36.61 (17.30)
Sex, *n* (%)	
Male	1041 (77.60)
Female	301 (22.40)
Marital Status, *n* (%)	
Never Married	710 (52.90)
Married	383 (28.50)
Divorced	122 (9.10)
Separated	71 (5.30)
Widowed	50 (3.70)
Other	3 (0.20)
Missing/Refused	3 (0.20)
Education, *n* (%)	
Less than High School Graduate	654 (48.70)
High School Diploma	287 (21.40)
Some College	95 (7.10)
Associates Degree	45 (3.40)
Less than Bachelor’s	88 (6.60)
Bachelor’s Degree	39 (2.90)
Some Graduate School	3 (0.20)
Master’s Degree	7 (0.50)
Less than Doctoral Degree	3 (0.20)
Doctoral Degree	6 (0.40)
Missing/Refused	115 (8.60)
Annual Earnings, *n* (%)	
$9999 or less	163 (12.10)
$10,000–$19,999	204 (15.20)
$20,000–$29,999	158 (11.80)
$30,000–$39,999	114 (8.50)
$40,000–$49,999	62 (4.60)
$50,000–$59,999	27 (2.00)
$60,000–$69,999	19 (1.40)
$70,000–$79,999	11 (0.80)
$80,000–$89,999	9 (0.70)
$90,000–$99,999	7 (0.50)
$100,000 or more	8 (0.60)
Missing/Refused	560 (41.70)
Employment at Injury, n (%)	
Not Employed	386 (28.80)
Employed	830 (61.80)
Missing/Refused	126 (9.40)
Insurance Type, *n* (%)	
Private Insurance	533 (39.70)
Public or No Insurance	784 (58.40)
Missing/Refused	25 (1.90)
Country of Birth, n (%)	
United States	535 (39.90)
Other than United States	578 (43.10)
Missing/Refused	229 (17.10)
Language Spoken at Home, *n* (%)	
Spanish	631 (47.00)
English	492 (36.70)
Missing/Refused	219 (16.30)
Cause of Injury, *n* (%)	
Violent	215 (16.10)
Non-Violent	1122 (83.60)
Missing/Refused	5 (0.40)
Days in PTA, *M* (*SD*)	27.41 (23.24)

**Table 2:** Demographic and injury predictors of average number of drinks per episode and five or more drinks in the previous month across 1, 2, 5, and 10 years.

	Ave. # of Drinks		5 or More Drinks	
Predictor	b-Weight	*p*-Value	b-Weight	*p*-Value
Intercept	−0.25	0.519	0.17	0.719
Time	−0.15	0.007	0.12	0.079
Time*Time	−0.01	0.152	−0.01	0.404
Average Number of Drinks Per Episode before Injury	0.14	<0.001	-	-
Five or More Drinks in Previous Month before Injury	-	-	0.09	<0.001
Age	−0.01	0.250	0.00	0.990
Sex (0 = woman, 1 = man)	0.35	0.033	0.52	0.008
Partnered (0 = single, 1 = partnered)	−0.31	0.046	−0.36	0.059
PTA ^1^	−0.01	<0.001	−0.00	0.236
Education	0.07	0.001	0.00	0.887
Employment at Injury (0 = not employed, 1 = employed)	0.30	0.288	−0.11	0.745
Earnings	−0.04	0.224	−0.04	0.298
Insurance Type (0 = other, 1 = private insurance)	−0.06	0.667	−0.13	0.429
Country of Origin (0 = U.S. 1 = Other)	0.05	0.771	0.18	0.368
Language Spoken at Home (0 = Other, 1 = English)	0.32	0.061	0.30	0.146
Cause of Injury (0 = non-violent, 1 = violent)	−0.57	0.001	−0.27	0.202

^1^ PTA = posttraumatic amnesia.

**Table 3:** Previously significant predictors and time interactions on average number of drinks per episode and five or more drinks in the previous month across 1, 2, 5, and 10 years.

	Average Number of Drinks		5 or More Drinks	
Predictor	b-Weight	*p*-Value	b-Weight	*p*-Value
Average Number of Drinks Per Episode before Injury HLM ^2^				
Intercept	0.82	<0.001	-	-
Time	0.16	<0.001	-	-
Time*Time	−0.01	0.009	-	-
Age	0.13	<0.001	-	-
Time*Age	−0.01	0.629	-	-
Time*Time*Age	0.00	0.078	-	-
5 or More Drinks Previous Month before Injury HLM ^2^				
Intercept	-	-	0.43	<0.001
Time	-	-	0.16	0.001
Time*Time	-	-	−0.01	0.008
5 or more drinks in month before injury	-	-	0.06	<0.001
Time*5 or more drinks in month before injury	-	-	0.04	<0.001
Time*Time*5 or more drinks in month before injury	-	-	−0.00	0.056
Sex HLM ^2^				
Intercept	−0.49	<0.001	0.18	0.204
Time	0.21	0.007	0.09	0.308
Time*Time	−0.02	0.024	−0.01	0.345
Sex (0 = woman, 1 = man)	0.43	<0.001	0.31	0.047
Time*Sex	−0.08	0.373	0.05	0.633
Time*Time*Sex	0.01	0.221	−0.00	0.994
Cause of Injury HLM ^2^				
Intercept	0.85	<0.001	-	-
Time	0.17	<0.001	-	-
Time*Time	−0.01	0.007	-	-
Cause of Injury (0 = Non-violent, 1 = Violent)	−0.11	0.450	-	-
Time*Cause of Injury	−0.15	0.117	-	-
Time*Time*Cause of Injury	0.02	0.115	-	-
Education HLM ^2^				
Intercept	0.40	0.016	-	-
Time	0.04	0.724	-	-
Time*Time	−0.01	0.660	-	-
Education	0.05	0.005	-	-
Time*Education	0.01	0.331	-	-
Time*Time*Education	−0.00	0.733	-	-
Partnered HLM ^2^				
Intercept	0.93	<0.001	-	-
Time	0.14	<0.001	-	-
Time*Time	−0.01	0.031	-	-
Partnered (0 = single, 1 = partnered)	−0.34	0.005	-	-
Time*Partnered	0.00	0.959	-	-
Time*Time*Partnered	0.00	0.841	-	-
PTA HLM ^2^				
Intercept	0.84	<0.001	-	-
Time	0.15	<0.001	-	-
Time*Time	−0.01	0.031	-	-
PTA ^1^	−0.01	0.003	-	-
Time*PTA ^1^	−0.00	0.315	-	-
Time*Time*PTA ^1^	0.00	0.334	-	-

^1^ PTA = posttraumatic amnesia; ^2^ HLM = Hierarchical linear modeling.

## Data Availability

Publicly available data were analyzed in this study. Information on access to these data can be found here: https://www.tbindsc.org/Researchers.aspx.
